# Humoral and cellular immune response and the safety of third SARS-CoV-2 mRNA vaccine with longer interval after the second vaccination in kidney transplant recipients

**DOI:** 10.3389/fimmu.2022.1050211

**Published:** 2022-11-30

**Authors:** Satoshi Takai, Hayato Nishida, Hiromi Ito, Hiroki Fukuhara, Takaaki Nawano, Takafumi Narisawa, Hidenori Kanno, Mayu Yagi, Atsushi Yamagishi, Toshihiko Sakurai, Sei Naito, Tomoyuki Kato, Keita Morikane, Norihiko Tsuchiya

**Affiliations:** ^1^ Department of Urology, Yamagata University Faculty of Medicine, Yamagata, Japan; ^2^ Department of Cardiology, Pulmonology, and Nephrology, Yamagata University Faculty of Medicine, Yamagata, Japan; ^3^ Division of Clinical Laboratory and Infection Control, Yamagata University Hospital, Yamagata, Japan

**Keywords:** kidney transplantation, rituximab, COVID-19, SARS-CoV-2, mRNA vaccine

## Abstract

We evaluated the humoral and cellular immune responses and safety of the third severe acute respiratory syndrome coronavirus 2 (SARS-CoV-2) mRNA vaccine with a longer interval after the second vaccination in kidney transplant recipients (KTRs). We enrolled 54 kidney transplant recipients without a history of coronavirus disease 2019 (COVID-19), who received a third dose of the vaccine. We assessed anti-SARS-CoV-2 spike antibody and antigen-specific T cells using enzyme-linked immunospot (ELISpot) against the spike protein at baseline, after the second vaccination, and after the third vaccination. We also evaluated the adverse events related to each dose of the vaccine. The duration between the second and third vaccinations was 7 ± 1 month. All 17 (100%) KTRs with anti-SARS-CoV-2 antibody positivity after the second vaccination and 27 of 37 (73%) KTRs without anti-SARS-CoV-2 antibody positivity after the second vaccination were positive for anti-SARS-CoV-2 antibodies (p=0.022). Anti-SARS-CoV-2 antibody titers were significantly higher than those after the second vaccination (p<0.001). Age ≥ 60 years and lymphocyte count < 1150/mm^3^ were confirmed as risk factors for anti-SARS-CoV-2 antibody negativity after the third vaccination in multivariate regression analysis. ELISpot cytokine activities were positive after the third vaccination in 26 of 29 (90%) KTRs with ELISpot cytokine activity positivity after the second vaccination and 12 of 24 (50%) KTRs without ELISpot cytokine activity after the second vaccination. The rate of change in cytokine activity after the third vaccination was significantly higher than that after the second vaccination (p<0.001). Only lymphocyte counts less than 1150/mm^3^ were confirmed as risk factors for ELISpot cytokine activity negativity in the multivariate regression analysis. Systemic adverse events classified as greater than moderate did not differ for each vaccine dose. None of the patients showed clinical symptoms of acute rejection. The third SARS-CoV-2 mRNA vaccine administration, with a longer interval after the second vaccination, improved humoral and cellular immune responses to SARS-CoV-2 mRNA vaccines without severe adverse effects in the KTRs.

## Introduction

Coronavirus disease 2019 (COVID-19) caused by severe acute respiratory syndrome coronavirus 2 (SARS-CoV-2) has continued to spread without convergence. Several vaccines for SARS-CoV-2, including mRNA vaccines, have been developed and have succeeded in reducing the mortality and severity of COVID-19 (1). Rate of seroconversion after 2 doses of mRNA vaccination in virus-naïve kidney transplant recipients was reported 2.5 to 48% which was much lower than immunocompetent individuals (2). Immune response to SARS-CoV-2 vaccines was not sufficient to protect immunosuppressed patients, including organ transplant recipients. Mortality and severity in those patients remained high compared to immunocompetent individuals (3–6).

After the clinical utility of the third dose of SARS-CoV-2 mRNA vaccine to protect against infection and prevent severe illness was reported, the third vaccination became a standardized vaccination strategy to protect people from COVID-19 (7). Several studies have reported that the administration of a third vaccine improved both humoral and cellular responses to SARS-CoV-2 even in transplant recipients (8–15). In these studies, transplant recipients received the third vaccination 1–3 months after the second vaccination, which was shorter than the recommended duration for the general population, because of poor response to the second vaccination in transplant recipients; therefore, the efficacy of the third vaccine administered relatively long after the second vaccine has not been fully investigated. The third vaccination was also implemented in Japan for all applicants who had received the second vaccination, and the duration between the second and third vaccinations was at least six months for all applicants, including transplant recipients. Therefore, we evaluated the humoral and cellular immune responses and safety of the third SARS-CoV-2 mRNA vaccine with a longer interval after the second vaccination in kidney transplant recipients (KTRs).

## Materials and methods

### Patients

Of the 58 KTRs who were enrolled in a study that evaluated the immunogenicity of two doses of SARS-CoV-2 mRNA in KTRs at our department, 54 KTRs were enrolled in this study (16). All participants completed three doses of the SARS-CoV-2 mRNA-1273 vaccine (Moderna) or BNT162b2 SARS-CoV-2 mRNA vaccine (Pfizer-BioNTech) between January and June 2022. This study was conducted in accordance with the principles outlined in the Declaration of Helsinki. All the participants provided written informed consent. The ethics committee of Yamagata University Faculty of Medicine approved the protocol for this research project (approval no. 2021-329).

### Blood sample collection

Blood samples were obtained within 2 weeks before the first dose, 2–4 weeks after the second dose, and 1–7 weeks after the third dose of the vaccine. Serum creatinine levels recorded on the day of blood sample collection were retrieved from the patient records.

### Anti-SARS-CoV-2 antibody detection

The blood samples were tested using an anti-SARS-CoV-2 S enzyme immunoassay (Elecsys anti-SARS-CoV-2 S RUO; Roche Diagnostics, Mannheim, Germany), which detects antibodies against the receptor-binding domain of the SARS-CoV-2 spike protein, according to the manufacturer’s instructions. Values below 0.8 U/mL were considered negative.

### ELISpot analysis

To analyze cellular responses, an ELISpot assay measuring interferon-gamma produced by specific SARS-CoV-2 T cells was performed as previously described (16). Briefly, PBMCs were isolated by specific gravity centrifugation using Ficall-Paque Premium (Cytiva, Tokyo, Japan), and cryopreserved until analysis. Stimulation was conducted with individual sequences containing 11 amino acids overlapping a 15-mer peptide pool derived from a peptide scan of the full-length sequence of the vaccine (BNT162b2), which encoded the receptor-binding domain of the SARS-CoV-2 spike glycoprotein (2 µg/mL/peptide; JPT Peptide Technologies, Berlin, Germany). All tests were performed in duplicate. Negative control wells lacked peptides, and positive control wells contained anti-CD3 monoclonal antibodies (1:1000; Mabtech, Stockholm, Sweden). Then, 2×10^5^ PBMCs per well were stimulated, placed in a plate pre-coated with anti-IFN-γ (Human INF-γ ELISpotPro kit, Mabtech, Stockholm, Sweden) at 37°C in a humidified incubator with 5% CO_2_, and incubated for 48 h. The cells were removed, and the plates were washed five times with phosphate-buffered saline. Next, 100 μL of a 200-fold diluted secondary anti-INF-γ antibody conjugated with horseradish peroxidase (ELISpotPro kit, Mabtech, Stockholm, Sweden) was added to each well and incubated at room temperature for 2 h. After five washes with phosphate-buffered saline, a tetramethylbenzidine substrate was added. ELISpot analysis was performed using an ELISpot Reader (Autoimmun Diagnostika; Strasberg, Germany). Cytokine activity was calculated from spot size and intensity values, as previously described (16, 17). The rate of change in cytokine activity in each test was calculated using the formula below, and the mean value of the two measurements was used as the measured value.

The rate of change in cytokine activity = 100 × (cytokine activity in peptide-stimulated wells − cytokine activity in negative control wells)/cytokine activity in negative control wells.

The cut-off value was determined by calculating the mean ± 2 standard deviations in a group of healthcare workers obtained prior to the first vaccination in our previous study, and was determined as the rate of change in cytokine activity greater than 164 (16).

### Vaccine safety

Adverse reactions after each dose of vaccine were documented using a specific questionnaire that included local reactions (pain, redness, and swelling at the injection site) and systemic reactions (fever, fatigue, headache, chills, myalgia, arthralgia, vomiting, and diarrhea). Participants were also asked to rate their symptoms on an ordinal scale (none, mild, moderate, or severe). Mild symptoms were defined as those that did not interfere with daily activities, moderate symptoms were defined as those that caused some interference with daily activities, and severe symptoms were defined as those that prevented daily activities.

Clinical episodes of acute rejection, including acute elevation in eGFR after the third vaccination, were also evaluated. eGFR was calculated using a formula modified for Japanese patients, as regulated by the Japanese Society of Nephrology (eGFR = 194 serum creatinine mg/dL^1.094^ × age^0.287^ × 0.739 [if female]).

### Statistical analysis

All clinical data were collected from patient records and analyzed retrospectively. Statistical analysis of various parameters was performed for each group using Fisher’s exact test for categorical variables and Mann–Whitney *U* or Wilcoxon signed-rank tests for continuous variables. The significance level was set at p < 0.05. All statistical analyses were performed using EZR (Saitama Medical Center, Jichi Medical University, Saitama, Japan), a graphical user interface that is a modified version of R commander designed to add statistical functions frequently used in biostatistics (The R Foundation for Statistical Computing, Vienna, Austria) (18).

## Results

### Demographic characteristics of the study participants

Of the 58 KTRs who were enrolled in the study that evaluated the immunogenicity of two doses of SARS-CoV-2 mRNA in KTRs at our department, 1 patient did not receive the third vaccine, 2 patients did not want to participate in the study, and 1 patient was infected with SARS-CoV-2 before the third vaccination. Finally, 54 KTRs who did not have a clinical episode of COVID-19 before the third vaccine were enrolled in this study. The clinical characteristics of the participants are shown in **Table 1**.

**Table 1:** Patient demographics.

		KTR
		(n=54)
Age, years (mean, SD)		54 (15)
Sex		
	Male (%)	37 (69)
First and second vaccine type		
	BNT162b2 (%)	53 (98)
	mRNA-1273 (%)	1 (2)
Third vaccine type		
	BNT162b2 (%)	28 (52)
	mRNA-1273 (%)	26 (48)
Time from KT to first vaccine, months (mean, SD)		83 (72)
Time from second to third vaccine, months (mean, SD)		7 (1)
Donor type		
	Living donor (%)	49 (91)
	Deceased donor (%)	5 (9)
Retransplantation (%)		4 (7)
ABO incompatible KT (%)		16 (30)
DSA positive before vaccine		7 (13)
IS medication		
	Steroid (%)	48 (89)
	Tacrolimus (%)	74 (91)
	Cyclosporine (%)	26 (9)
	MMF with short-term EVL conversion (%)	17 (31)
	MMF without short-term EVL conversion (%)	29 (54)
	EVL excluding short-term conversion (%)	9 (17)
	Mizoribine (%)	2 (4)
	Rituximab (%)	21 (39)
Comorbidities		
	Hypertension (%)	20 (37)
	Diabetes (%)	18 (33)
	Cardiovascular diseases (%)	8 (15)
	History of malignancy (%)	9 (17)
eGFR, mL/min/1.73m^2^, (mean, SD)		52 (18)
SARS-CoV-2 anti-spike antibody positive after second vaccine (%)		17 (31)
ELISpot activity positive after second vaccine (%)		29 (54)

DSA, donor specific anti-human leukocyte antigen antibody; eGFR, estimated glomerular filtration rate; IS, immunosuppression; EVL, everolimus; KT, kidney transplantation; MMF, mycophenolate mofetil; RIT, rituximab; SD, standard deviation; UP/C, urine protein to creatinine ratio.

In 53 of 54 (98%) KTRs who received the BNT162b2 vaccine as the first and second vaccine, 28 (53%) received the BNT162b2 vaccine and 25 (47%) received the mRNA-1273 vaccine as the third vaccine. Only 1 of 54 (2%) KTRs who received mRNA-1273 as the first and second vaccine received the mRNA-1273 vaccine as the third vaccine. The duration between the second and third vaccinations was 7 ± 1 month. No significant difference between KTRs with BNT162b2 as third vaccine and mRNA-1273 as third vaccine was observed in the positivity rate of the anti-SARS-CoV-2 antibody (KTRs with BNT162b2, 32%; KTRs with mRNA-1273, 31%; p=1.000), anti-SARS-CoV-2 antibody titer (KTRs with BNT162b2, 83 ± 296 U/mL; KTRs with mRNA-1273, 108 ± 442 U/mL; p=0.749), the positivity rate of ELISpot activity (KTRs with BNT162b2, 50%; KTRs with mRNA-1273, 58%; p=0.597), and the rate of change in ELISpot cytokine activity (KTRs with BNT162b2, 386 ± 572; KTRs with mRNA-1273, 240 ± 238; p=0.545) after the second vaccination. Forty-nine of 54 (91%) were living donor KTRs, and 16 of 54 (30%) KTRs received ABO-incompatible kidney transplants (KT). All KTRs were treated with calcineurin inhibitors, and 48 of 54 (89%) KTRs were treated with steroids. Seventeen KTRs were converted from mycophenolate mofetil (MMF) to everolimus (EVL) (0.75 mg bid) only one week before and after the third vaccination in 46 of 54 KTRs who had been treated with MMF. All 21 KTRs with rituximab received rituximab only for the induction of ABO-incompatible and/or sensitized KT. There were 6 patients who received rituximab within 24 months before the first vaccination. The duration between the rituximab induction and the first vaccinations was 61 ± 41 month. Only 17 of 54 KTRs (31%) were SARS-CoV-2 anti-spike antibody-positive, and 29 of 54 (54%) were ELISpot activity-positive after the second vaccination.

### Anti-SARS-CoV-2 antibody levels after the third vaccination

**Figure 1:**
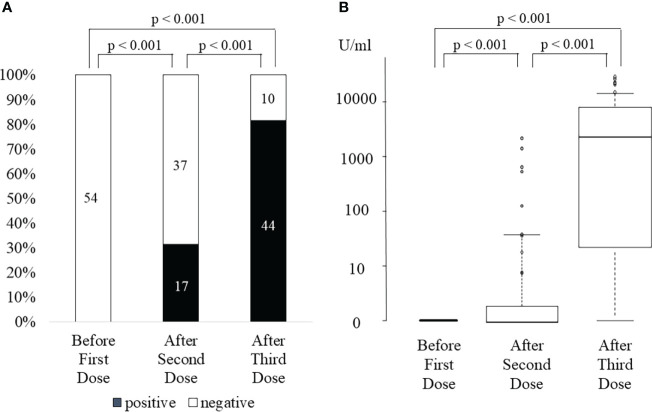
SARS-CoV-2 anti-spike antibody positivity rate **(A)** and antibody titer **(B)** before first dose, after the second dose, and after the third dose of SARS-CoV-2 mRNA vaccine.

After the third vaccination, all 17 (100%) KTRs with anti-SARS-CoV-2 antibody positivity after the second vaccination and 27 of 37 (73%) KTRs without anti-SARS-CoV-2 antibodies after the second vaccination were positive for anti-SARS-CoV-2 antibodies (**Figure 1A**). The anti-SARS-CoV-2 antibody seroconversion rate after the third vaccination was 81.4%, which was higher than that reported in previous studies (6.4-69.2%) (**Table 2**) (8, 10–15). Moreover, anti-SARS-CoV-2 antibody titers were significantly higher than those after the second vaccination not only in all KTRs (2^nd^, 95 ± 370 U/mL; 3^rd^, 6673 ± 10676 U/mL; p<0.001) but also in KTRs with anti-SARS-CoV-2 antibody positivity after the second vaccination (2^nd^, 301 ± 622 U/mL; 3^rd^, 15385 ± 14173 U/mL; p<0.001) (**Figure 1B**). Both neutrophil counts and IgG titers before the third vaccination did not differ between KTRs with anti-SARS-CoV-2 antibody and those without it after the third vaccination; however, lymphocyte count was significantly lower in KTRs without anti-SARS-CoV-2 antibody after the third vaccination (p=0.006). In the univariate model, age ≥ 60 years, first vaccination within 2 years after kidney transplantation, and lymphocyte count < 1150/mm^3^ were associated with a lack of humoral response to the third vaccination (**Table 3**). Multivariate regression analysis, accounting for age ≥ 60 years and lymphocyte count < 1150/mm^3^, confirmed these associations. Rituximab did not affect anti-SARS-CoV-2 antibody positivity; however, anti-SARS-CoV-2 antibody was not detected in 4 of 6 KTRs who received rituximab within 24 months before the first vaccination. Short-term peri-vaccination conversion from MMF to EVL did not improve anti-SARS-CoV-2 antibody positivity. MMF, which was one of the risk factors for a lack of humoral response to the second vaccination, did not significantly affect the anti-SARS-CoV-2 antibody positivity rate after the third vaccination. In contrast, the anti-SARS-CoV-2 antibody titer was significantly lower in KTRs with MMF (KTRs with MMF, 4853 ± 8407 U/mL; KTRs without MMF, 17141 ± 16174 U/mL; p=0.016).

**Table 2:** Rate of positivity after the third vaccination of SARS-CoV-2 mRNA vaccine.

Study population	Vaccine type	Time from second to third vaccine	Anti-SARS-CoV-2anti-spike antibody positiveafter third vaccine	Reference
54 KTRs	BNT162b2 or	231 days (average)	81.4%	Our study
	mRNA-1273			
30 SOTRs	BNT162b2 or	67 days (median) (IQR, 54 to 81 days)	47%	8
	mRNA-1273 or			
	ad26.COV2.S			
159 KTRs	mRNA-1273	51 days (median) (IQR, 27 to 33 days)	49%	10
396 SOTRs	BNT162b2	59 days (median) (IQR, 47 to 67 days)	67.9%	11
62 KTRs	BNT162b2	69.5 days (median) (IQR, 16.5 to 51.7 days)	6.4%	12
with belatacept				
456 KTRs	BNT162b2	50 days (average)	69.2%	13
71 KTRs	BNT162b2	68 days (average)	55%	14
101 SOTRs	BNT162b2	61 days (average)	68%	15

IQR, interquartile range; KTRs, kidney transplant recipients; SOTRs, solid organ transplant recipients.

**Table 3:** Factors associated with a negative antibody response after the third vaccination in kidney transplant recipients.

	Univariate			Multivariate		
	OR	95% CI	p-value	OR	95% CI	p-value
Demographics						
Age>= 60 years	15.75	1.82-135.87	0.012	14.10	1.49-134.04	0.021
Male (ref: female)	1.09	0.24-4.85	0.911			
Body mass index >=25	0.70	0.08-6.58	0.758			
Third vaccine type						
BNT162b2 (ref: mRNA-1273)	1.10	0.28-4.33	0.897			
Transplantation characteristics						
KT<2 year	6.34	1.40-28.65	0.017	6.08	0.82-44.90	0.077
Living donor (ref: deceased donor)	9.17×10^-8^	0-Inf	0.993	9.00×10^6^	0-Inf	0.994
Retransplantation	1.52	0.14-16.30	0.730			
ABO incompatible	3.00	0.73-12.30	0.128			
DSA positive	4.29	0.78-23.40	0.093			
IS medication						
Steroid	1.12×10^7^	0-Inf	0.992	2.84×10^7^	0-Inf	0.994
Tacrolimus (ref: cyclosporin)	0.50	0.06-4.52	0.538			
MMF	1.70	0.19-15.70	0.638			
EVL	1.32	0.23-7.59	0.755			
Rituximab	2.90	0.71-11.90	0.139			
MMF to EVL conversion	0.48	0.09-2.57	0.394			
Comorbidities						
Hypertension	1.47	0.33-6.47	0.611			
Diabetes	1.43	0.35-5.88	0.621			
eGFR<60ml/min/1.73m^2^	1.50	0.28-8.09	0.637			
Cardiovascular diseases	1.58	0.27-9.32	0.611			
History of malignancy	1.32	0.23-7.59	0.755			
Lymphocytes<1150/mm^3^	9.07	1.95-42.20	0.005	8.07	1.48-43.90	0.016

CI, confidence interval; Inf, infinity; DSA, donor specific anti-human leukocyte antigen antibody; eGFR, estimated glomerular filtration rate; EVL, everolimus; IS, immunosuppression; KT, kidney transplantation; MMF, mycophenolate mofetil; OR, odds ratio; ref, reference.

### Cellular response after the third vaccination

**Figure 2:**
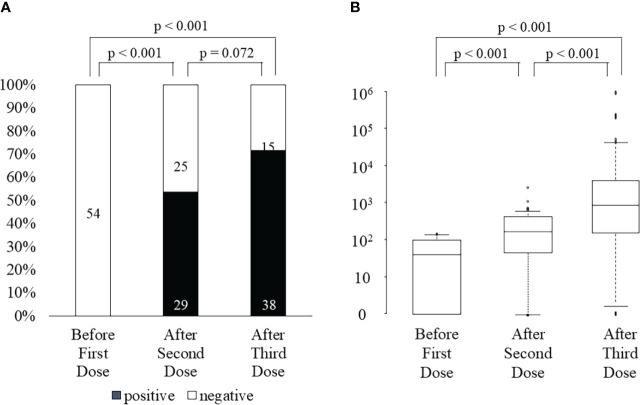
ELISpot activity positivity rate **(A)** and the rate change in cytokine activity **(B)** before first dose, after the second dose, and after the third dose of SARS-CoV-2 mRNA vaccine.

After the third vaccination, 26 of 29 (90%) KTRs with positive ELISpot cytokine activity after the second vaccination, and 12 of 24 (50%) KTRs without ELISpot cytokine activity after the second vaccination were positive for ELISpot cytokine activity (**Figure 2A**). All three KTRs whose ELISpot cytokine activity turned negative after the third vaccination received triple immunosuppressants consisting of steroids, MMF, and tacrolimus and the doses of these immunosuppressants did not change during the study period. Their lymphocyte counts did not change significantly. Anti-SARS-CoV-2 antibody test was negative after the second vaccination in all three KTRs; however, the anti-SARS-CoV-2 antibody test was positive after the third vaccination (22, 7913, and 11167 U/mL). The rate of change in cytokine activity after the third vaccination was significantly higher than that after the second vaccination, not only in all KTRs (3^rd^, 34690 ± 133690; 2^nd^, 316 ± 445; p<0.001) but also in KTRs without ELISpot cytokine activity after the second vaccination (2^nd^,554 ± 496; 3^rd^, 58932 ± 178090; p<0.001) (**Figure 2B**). Neither neutrophil counts, lymphocyte counts, nor IgG titers differed between KTRs with anti-SARS-CoV-2 antibody positivity and those without it after the third vaccination. In univariate analysis, hypertension and lymphocyte count < 1150/mm^3^ were associated with a lack of positive ELISpot activity after the third vaccination (**Table 4**). Multivariate regression analysis, accounting for lymphocytes less than 1150/mm^3^, confirmed this association.

**Table 4:** Factors associated with a negative cellular response after the third vaccination in kidney transplant recipients.

	Univariate			Multivariate		
	OR	95% CI	p-value	OR	95% CI	p-value
Demographics						
Age>=60 years	3.07	0.87-10.80	0.080			
Male (ref: female)	0.43	0.10-1.78	0.244			
Body mass index >=25	0.38	0.04-3.47	0.392			
Third vaccine type						
BNT162b2 (ref: mRNA-1273)	0.97	0.29-3.22	0.963			
Transplantation characteristics						
KT<2 year	3.30	0.79-13.80	0.101			
Living donor (ref: deceased donor)	1.65	0.17-16.10	0.668			
Retransplantation	0.66	0.06-7.14	0.730			
ABO incompatible	0.35	0.10-1.25	0.107			
DSA positive	1.02	0.18-5.91	0.986			
IS medication						
Steroid	1.99×10^7^	0-Inf	0.992			
Tacrolimus (ref: cyclosporin)	0.68	0.12-3.73	0.658			
MMF	1.22	0.22-6.84	0.822			
EVL	0.68	0.13-3.73	0.658			
Rituximab	2.20	0.65-7.41	0.204			
MMF to EVL conversion	1.22	0.22-6.92	0.823			
Comorbidities						
Hypertension	5.26	1.04-26.60	0.045	3.77	0.70-20.28	0.122
Diabetes	1.44	0.42-4.99	0.561			
eGFR<60ml/min/1.73m^2^	7.30	0.86-61.72	0.069			
Cardiovascular diseases	3.09	0.66-14.50	0.152			
History of malignancy	0.32	0.04-2.82	0.303			
Lymphocytes<1150/mm^3^	9.07	1.95-42.20	0.005	5.06	1.37-18.70	0.015

CI, confidence interval; Inf, infinity; DSA, donor specific anti-human leukocyte antigen antibody; eGFR, estimated glomerular filtration rate; EVL, everolimus; IS, immunosuppression; KT, kidney transplantation; MMF, mycophenolate mofetil; OR, odds ratio; ref, reference.

### Correlation between humoral and cellular responses

In 44 KTRs who were anti-SARS-CoV-2 antibody-positive after the third vaccination, 28 (64%) KTRs were also ELISpot cytokine activity-positive. Neither humoral nor cellular responses after the third vaccination were observed in 9 of 54 (17%) KTRs. Only one KTR was ELISpot cytokine activity-positive but anti-SARS-CoV-2 antibody negative. In 44 KTRs with anti-SARS-CoV-2 antibody positivity after the third vaccination, the anti-SARS-CoV-2 antibody titer in KTRs with positive ELISpot cytokine activity was significantly higher than that in KTRs without ELISpot cytokine activity (9547 ± 12179 U/mL vs. 2909 ± 4127 U/mL, p=0.047).

### Adverse reactions after vaccination

**Figure 3:**
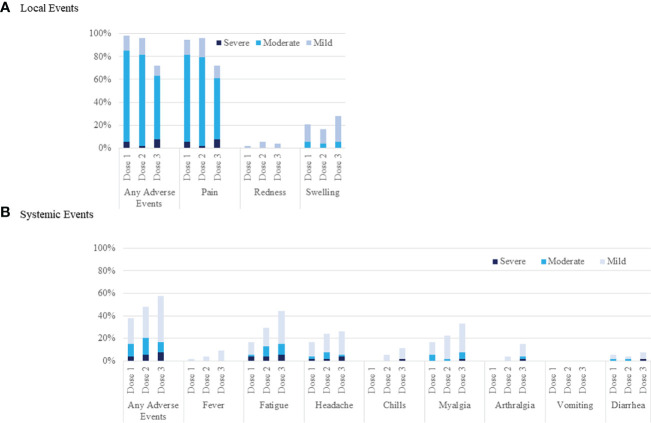
Local and systemic reactions before the first, after the second, and after the third injection of SARS-CoV-2 mRNA vaccine. Injection-site reactions are shown in panel **(A)**. Systemic events are shown in panel **(B)**.

Adverse events at the injection site after the third vaccination (72%) were significantly lower than those after both the first (96%, p<0.001) and second vaccinations (96%, p<0.001) (**Figure 3A**). Systemic adverse events after the third vaccination were not significantly higher (57%) than those after both the first (37%, p=0.053) and second vaccinations (48%, p=0.441) (**Figure 3B**). Systemic adverse events classified as greater than moderate did not differ between the first (15%), second (17%), and third vaccinations (17%) (p=1.000). Systemic adverse events classified as greater than moderate after the third vaccination were not correlated with anti-SARS-CoV-2 antibody positivity after the third vaccination (positive, 16%; negative, 20%; p=1.000), ELISpot cytokine activity positivity after the third vaccination (positive, 16%; negative, 20%; p=0.701), or third vaccine type (BNT162b2 vaccine, 20%; mRNA-1732 vaccine, 14%; p=0.719). There was no significant deterioration of the eGFR after the third vaccination (2^nd^, 52.1 ± 18.1 mL/min/1.73 m^2^; 3^rd^, 52.6 ± 18.5 mL/min/1.73 m^2^; p=0.926). None of the patients showed clinical symptoms of acute rejection.

## Discussion

Consistent with the poor humoral and cellular responses to two doses of SARS-CoV-2 mRNA vaccine in organ transplant recipients, several studies have reported insufficient protective effects against both SARS-CoV-2 infection and severe illness (3–5, 19, 20). As reported in several studies that demonstrated improved immunogenicity after the third vaccination with shorter periods from the second vaccination, we confirmed improved humoral and cellular responses for SARS-CoV-2 and safety profiles after the third vaccination, even with relatively long periods from the second vaccination.

We confirmed a higher anti-SARS-CoV-2 antibody positive rate and higher titer of anti-SARS-CoV-2 antibody after the third vaccination than after the second vaccination in KTRs with anti-SARS-CoV-2 antibody positivity after the second vaccination. We also confirmed a high anti-SARS-CoV-2 antibody seroconversion rate (73%) after the third vaccination in KTRs without anti-SARS-CoV-2 antibodies after the second vaccination. The anti-SARS-CoV-2 antibody seroconversion rate after the third vaccination in organ transplant recipients who were negative before the third vaccination was reported as 6–45%, which was lower than that in our study (8, 10–14). Although it was not a study on mRNA vaccines but on adenoviral vectored vaccines, a higher protective efficacy according to higher binding and neutralizing antibody titers in those with a longer interval from the first to second vaccination was reported in the ChAdOx1 nCoV-19 vaccine trial (21). Greater protective efficacy associated with stronger immune responses after a wider interval between the first and second vaccinations has also been demonstrated with other vaccines, such as those for influenza, Ebola virus disease, and malaria (22–24). It was suggested that taking the same interval from the second to the third vaccination as the general population, might improve vaccine immunogenicity, rather than the third dose administered with a shorter interval for organ transplant recipients without sufficient immunogenicity after the second vaccination.

In previous studies, immunosuppressants, including steroids, MMF, belatacept, and triple immunosuppression; elderly patients; short period from transplantation; low lymphocyte counts; and low allograft function were confirmed as risk factors for negative immunogenicity after the third vaccination (8, 10–15). Age > 60 years, low lymphocyte counts, and transplantation within 2 years were identified as risk factors in our study; however, immunosuppressants, including MMF, did not affect the anti-SARS-CoV-2 antibody positivity rate in our study, even though MMF was confirmed as a risk factor for a negative humoral response after the second SARS-CoV-2 mRNA vaccine in our previous study (16). MMF did not affect the seropositivity rate; however, the anti-SARS-CoV-2 antibody titer was significantly lower in KTRs with MMF. This means that the third vaccination could induce antibody production even in KTRs with MMF, but this was not sufficient. Our results showed that the anti-SARS-CoV-2 antibody titer was much higher after the third vaccination in all KTRs with MMF and anti-SARS-CoV-2 antibody-positive after the second vaccination (2^nd^; 75 ± 331 U/ml, 3^rd^; 5912 ± 9219 U/ml, p<0.001); elevation of antibody titer due to additional immune stimulation by the fourth vaccination will be expected in KTRs with MMF.

Both the ELISpot cytokine activity positivity rate and the rate of change in cytokine activity were improved by the third vaccination; however, we observed three KTRs whose ELISpot cytokine activity turned negative after the third vaccination. Several studies have reported improved cellular reaction after the third vaccination compared with that after the second vaccination or that in the placebo group in organ transplant recipients (9, 25, 26). In contrast, Stumpf J et al. reported a decreased cellular response rate after the third vaccination compared with that after the second vaccination (14). The change in cellular immune response after the third vaccination in each patient was not described in those studies; therefore, the differences in patient characteristics between patients with an enhanced cellular immune response and those with an attenuated response were unclear. There were no specific characteristics in the three KTRs whose ELISpot cytokine activity turned negative, and none of these KTRs received an intervention that enhanced immunosuppression between the second and third vaccinations. Further investigation is necessary to determine the cause of this phenomenon.

Our study has several limitations. First, the number of vaccinated participants was low. Second, we examined both humoral and cellular reactions only once after the second and third vaccinations. Ben-Dov IZ et al. reported an improvement in the anti-SARS-CoV-2 antibody positivity rate over time after the second vaccination (27). The timing of the blood sample collection might have affected the results of this study. Third, we could not evaluate formal neutralization against variants of concern, including the Omicron subvariants BA.4 and BA.5. Fourth, we could not evaluate the protective effect of vaccination against SARS-CoV-2 infection because of the low incidence of actual COVID-19 in this cohort and the high efficacy of newly developed antiviral therapies including monoclonal antibodies, nirmatrelvir/ritonavir, molnupiravir, and remdesivir for patients with risk factors. Finally, the fourth vaccination for candidates with risk factors for severe COVID-19, including KTRs, has been initiated, and several studies have reported improved antibody response (28–30). There were KTRs who could not experience anti-SARS-CoV-2 antibody seroconversion and/or cellular responses in this cohort, so the fourth vaccination was necessary; therefore, it should also be evaluated whether the immunogenicity is improved.

In conclusion, the third SARS-CoV-2 mRNA vaccine with a longer interval after the second vaccination might improve humoral and cellular immune responses to SARS-CoV-2 mRNA vaccines without severe adverse effects in the KTRs. While in the beginning of the pandemic rapid immunization of the risk group was critical, we now should try to identify the best vaccination schedules to provide long term protection and variant cross-recognition. Prospective randomized control study with different dose intervals is needed to conclude this information. Evaluation of the protective effect against both SARS-CoV-2 infection and severe illness by the third vaccination and immunogenicity of the fourth vaccination should be studied in a larger cohort in the future.

## Data availability statement

The raw data supporting the conclusions of this article will be made available by the authors, without undue reservation.

## Ethics statement

The studies involving human participants were reviewed and approved by The ethics committee of Yamagata University Faculty of Medicine. The patients/participants provided their written informed consent to participate in this study.

## Author contributions

ST, HN, and HI were involved in research design, data acquisition, data analysis, interpretation, and writing of the manuscript. HF, TNaw, TNar, HK, MY, AY, TS, SN, and KM were involved in data analysis and review of the manuscript. NT was involved in research design, data analysis, and review of the manuscript. All authors contributed to the article and approved the submitted version.

## Acknowledgments

The authors would like to thank the medical technologists for technical assistance with the experiments and the medical staff for collecting clinical samples. We would like to thank Editage (www.editage.com) for English language editing.

## Conflict of interest

The authors declare that the research was conducted in the absence of any commercial or financial relationships that could be construed as a potential conflict of interest.

## Publisher’s note

All claims expressed in this article are solely those of the authors and do not necessarily represent those of their affiliated organizations, or those of the publisher, the editors and the reviewers. Any product that may be evaluated in this article, or claim that may be made by its manufacturer, is not guaranteed or endorsed by the publisher.

## References

[1] Watson OJ Barnsley G Toor J Hogan AB Winskill P Ghani AC. Global impact of the first year of COVID-19 vaccination: A mathematical modelling study. Lancet Infect Dis (2022) 22:1293–302. doi: 10.1016/S1473-3099(22)00320-6 PMC9225255 35753318

[2] Caillard S Thaunat O. COVID-19 vaccination in kidney transplant recipients. Nat Rev Nephrol (2021) 17:785–7. doi: 10.1038/s41581-021-00491-7 PMC8475856 34580488

[3] Bell S Campbell J Lambourg E Watters C O'Neil M Almond A. The impact of vaccination on incidence and outcomes of SARS-CoV-2 infection in patients with kidney failure in Scotland. J Am Soc Nephrol (2022) 33:677–86. doi: 10.1681/ASN.2022010046 PMC8970454 35110363

[4] Qin CX Moore LW Anjan S Rahamimov R Sifri CD Ali NM. Risk of breakthrough SARS-CoV-2 infections in adult transplant recipients. Transplantation (2021) 105:e265–e6. doi: 10.1097/TP.0000000000003907 PMC8549120 34310531

[5] Mazuecos A Villanego F Zarraga S Lopez V Oppenheimer F Llinas-Mallol L. Breakthrough infections following mRNA SARS-CoV-2 vaccination in kidney transplant recipients. Transplantation (2022) 106:1430–9. doi: 10.1097/TP.0000000000004119 PMC9213063 35384924

[6] Caillard S Chavarot N Bertrand D Kamar N Thaunat O Moal V. Occurrence of severe COVID-19 in vaccinated transplant patients. Kidney Int (2021) 100:477–9. doi: 10.1016/j.kint.2021.05.011 PMC8141267 34029554

[7] Bar-On YM Goldberg Y Mandel M Bodenheimer O Freedman L Kalkstein N. Protection of BNT162b2 vaccine booster against covid-19 in Israel. N Engl J Med (2021) 385:1393–400. doi: 10.1056/NEJMoa2114255 PMC8461568 34525275

[8] Werbel WA Boyarsky BJ Ou MT Massie AB Tobian AAR Garonzik-Wang JM. Safety and immunogenicity of a third dose of SARS-CoV-2 vaccine in solid organ transplant recipients: a case series. Ann Intern Med (2021) 174:1330–2. doi: 10.7326/L21-0282 PMC8252023 34125572

[9] Hall VG Ferreira VH Ku T Ierullo M Majchrzak-Kita B Chaparro C. Randomized trial of a third dose of mRNA-1273 vaccine in transplant recipients. N Engl J Med (2021) 385:1244–6. doi: 10.1056/NEJMc2111462 PMC8385563 34379917

[10] Benotmane I Gautier G Perrin P Olagne J Cognard N Fafi-Kremer S. Antibody response after a third dose of the mRNA-1273 SARS-CoV-2 vaccine in kidney transplant recipients with minimal serologic response to 2 doses. JAMA (2021) 326:1063–5. doi: 10.1001/jama.2021.12339 PMC8456389 34297036

[11] Del Bello A Abravanel F Marion O Couat C Esposito L Lavayssiere L. Efficiency of a boost with a third dose of anti-SARS-CoV-2 messenger RNA-based vaccines in solid organ transplant recipients. Am J Transplant (2022) 22:322–3. doi: 10.1111/ajt.16775 PMC8441706 34331842

[12] Chavarot N Morel A Leruez-Ville M Vilain E Divard G Burger C. Weak antibody response to three doses of mRNA vaccine in kidney transplant recipients treated with belatacept. Am J Transplant (2021) 21:4043–51. doi: 10.1111/ajt.16814 PMC9906354 34431207

[13] Masset C Kerleau C Garandeau C Ville S Cantarovich D Hourmant M. A third injection of the BNT162b2 mRNA COVID-19 vaccine in kidney transplant recipients improves the humoral immune response. Kidney Int (2021) 100:1132–5. doi: 10.1016/j.kint.2021.08.017 PMC8404389 34474075

[14] Stumpf J Tonnus W Paliege A Rettig R Steglich A Gembardt F. Cellular and humoral immune responses after 3 doses of BNT162b2 mRNA SARS-CoV-2 vaccine in kidney transplant. Transplantation (2021) 105:e267–e9. doi: 10.1097/TP.0000000000003903 PMC8549130 34342963

[15] Kamar N Abravanel F Marion O Couat C Izopet J Del Bello A. Three doses of an mRNA covid-19 vaccine in solid-organ transplant recipients. N Engl J Med (2021) 385:661–2. doi: 10.1056/NEJMc2108861 PMC8262620 34161700

[16] Takai S Nishida H Ito H Fukuhara H Nawano T Narisawa T. Immunogenicity and safety of two doses of SARS-CoV-2 mRNA vaccine in kidney transplant recipients with low-dose rituximab. Int J Urol (2022) 29:1279–86. doi: 10.1111/iju.14978 PMC9349512 35863901

[17] Maeda C Iizuka A Miyata H Kondou R Ashizawa T Kanematsu A. Alternative evaluation of an ELISPOT assay using cytokine activity as a novel parameter. Anticancer Res (2021) 41:3825–31. doi: 10.21873/anticanres.15175 34281842

[18] Kanda Y. Investigation of the freely available easy-to-use software 'EZR' for medical statistics. Bone Marrow Transplant (2013) 48:452–8. doi: 10.1038/bmt.2012.244 PMC3590441 23208313

[19] McEvoy CM Lee A Misra PS Lebovic G Wald R Yuen DA. Real-world impact of 2-dose SARS-CoV-2 vaccination in kidney transplant recipients. Transplantation (2022) 106:e279–e80. doi: 10.1097/TP.0000000000004081 PMC9038233 35220385

[20] Williams SV Whitaker HJ Mumford L Callaghan C Curtis RMK Stowe J. Effectiveness of COVID-19 vaccines against hospitalization and death with the SARS-CoV-2 delta variant in solid organ and islet transplant recipients. Transplantation (2022) 106:e310–e1. doi: 10.1097/TP.0000000000004104 PMC9128400 35283455

[21] Voysey M Costa Clemens SA Madhi SA Weckx LY Folegatti PM Aley PK. Single-dose administration and the influence of the timing of the booster dose on immunogenicity and efficacy of ChAdOx1 nCoV-19 (AZD1222) vaccine: A pooled analysis of four randomised trials. Lancet (2021) 397:881–91. doi: 10.1016/S0140-6736(21)00432-3 PMC7894131 33617777

[22] Ewer K Rampling T Venkatraman N Bowyer G Wright D Lambe T. A monovalent chimpanzee adenovirus Ebola vaccine boosted with MVA. N Engl J Med (2016) 374:1635–46. doi: 10.1056/NEJMoa1411627 PMC5798586 25629663

[23] Ledgerwood JE Zephir K Hu Z Wei CJ Chang L Enama ME. Prime-boost interval matters: A randomized phase 1 study to identify the minimum interval necessary to observe the H5 DNA influenza vaccine priming effect. J Infect Dis (2013) 208:418–22. doi: 10.1093/infdis/jit180 PMC3699006 23633407

[24] Fernandez-Arias C Arias CF Zhang M Herrero MA Acosta FJ Tsuji M. Modeling the effect of boost timing in murine irradiated sporozoite prime-boost vaccines. PloS One (2018) 13:e0190940. doi: 10.1371/journal.pone.0190940 29329308 PMC5766151

[25] Bertrand D Hamzaoui M Lemee V Lamulle J Laurent C Etienne I. Antibody and T-cell response to a third dose of SARS-CoV-2 mRNA BNT162b2 vaccine in kidney transplant recipients. Kidney Int (2021) 100:1337–40. doi: 10.1016/j.kint.2021.09.014 PMC8489274 34619232

[26] Yahav D Rahamimov R Mashraki T Ben-Dor N Steinmetz T Agur T. Immune response to third dose BNT162b2 COVID-19 vaccine among kidney transplant recipients-a prospective study. Transpl Int (2022) 35:10204. doi: 10.3389/ti.2022.10204 35529596 PMC9068869

[27] Ben-Dov IZ Oster Y Tzukert K Alster T Bader R Israeli R. Impact of tozinameran (BNT162b2) mRNA vaccine on kidney transplant and chronic dialysis patients: 3-5 months follow-up. J Nephrol (2022) 35:153–64. doi: 10.1007/s40620-021-01210-y PMC8731189 34988942

[28] Alejo JL Mitchell J Chiang TP Abedon AT Boyarsky BJ Avery RK. Antibody response to a fourth dose of a SARS-CoV-2 vaccine in solid organ transplant recipients: A case series. Transplantation (2021) 105:e280–e1. doi: 10.1097/TP.0000000000003934 PMC8612849 34428188

[29] Caillard S Thaunat O Benotmane I Masset C Blancho G. Antibody response to a fourth messenger RNA COVID-19 vaccine dose in kidney transplant recipients: A case series. Ann Intern Med (2022) 175:455–6. doi: 10.7326/L21-0598 PMC8754215 35007148

[30] Mitchell J Alejo JL Chiang TPY Kim J Chang A Abedon AT. Antibody response to a fourth dose of SARS-CoV-2 vaccine in solid organ transplant recipients: An update. Transplantation (2022) 106:e338–e40. doi: 10.1097/TP.0000000000004137 PMC9213059 35426888

